# Correlation between in vivo imaging and post‑mortem autopsy findings for the medico-legal evaluation of a right hepatic artery pseudoaneurysm as a fatal complication of laparoscopic cholecystectomy

**DOI:** 10.1007/s12024-024-00937-x

**Published:** 2025-01-11

**Authors:** Guido Pelletti, Maria Sech, Nicola Montanari, Emilio Quaia, Domenico Garcea, Susi Pelotti

**Affiliations:** 1 Unit of Legal Medicine, Department of Medical and Surgical Sciences, University of Bologna, Via Irnerio 49, 40126 Bologna, Italy; 2 Department of Radiology, University of Padua, 35138 Padua, Italy; 3 Ospedali Privati Forlì S.p.A., Via C. Versari, 1, 47121 Forlì, Italy

**Keywords:** Cholecystectomy, Intraoperative bleeding, Right hepatic artery pseudoaneurysm, Post-mortem CT, Autopsy

## Abstract

A 36-year-old woman diagnosed with complicated cholecystolithiasis underwent elective laparoscopic cholecystectomy (LC), then converted to open cholecystectomy because of a massive intraoperative bleeding. Hemostasis was performed with clipping and suturing the source of bleeding. In post-operative period, the patient suffered from persistent anemia associated with hemoperitoneum diagnosed through abdominal CT scanning, in absence of any sign of active bleeding. She died 16 days after the surgical procedure. Autopsy revealed the presence of 2 clips adjacent to the suture used for ligating the cystic artery and the presence of 3 surgical metal clips on the right hepatic artery, that should not be present in a routine cholecystectomy. The review of CT scans performed during the hospital stay revealed contrast extravasation from the right hepatic artery, near the 3 clips, allowing the post-mortem diagnosis of pseudoaneurysm (PA). The diagnosis of PA of right hepatic artery is typically made in living patients, through imaging, and autoptic identification is rarely obtained. The innovation of this report is to present an iconographic correlation between in vivo imaging and autopsy data, allowing for the tracing of the PA’s origin to the wall weakening caused by the placement of 3 clips on the right hepatic artery, and having significant medico-legal implications.

## Case report

**Fig. 1:**
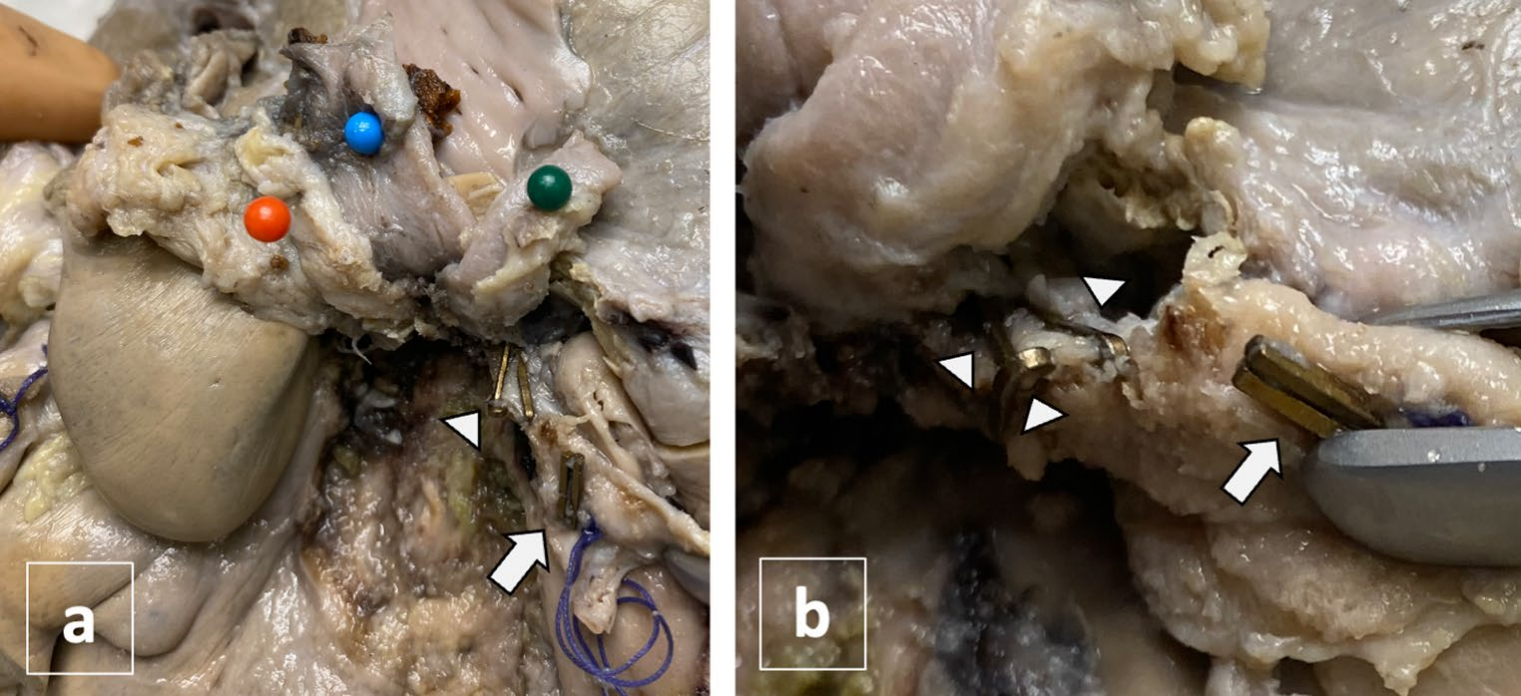
Macroscopic examination of the hepatic hilum after formalin fixation: hepatic hilum structures were elevated to visualize right hepatic artery. Portal vein was pinned in blue, left hepatic artery in orange and biliary tract in green (**a**). On right hepatic artery were identified 5 metal clips: 2 clips were located at the origin of cystic artery (white arrow) together with a surgical wire and 3 clips were on the distal segment, according to blood flow (arrow points) (**b**)

**Fig. 2:**
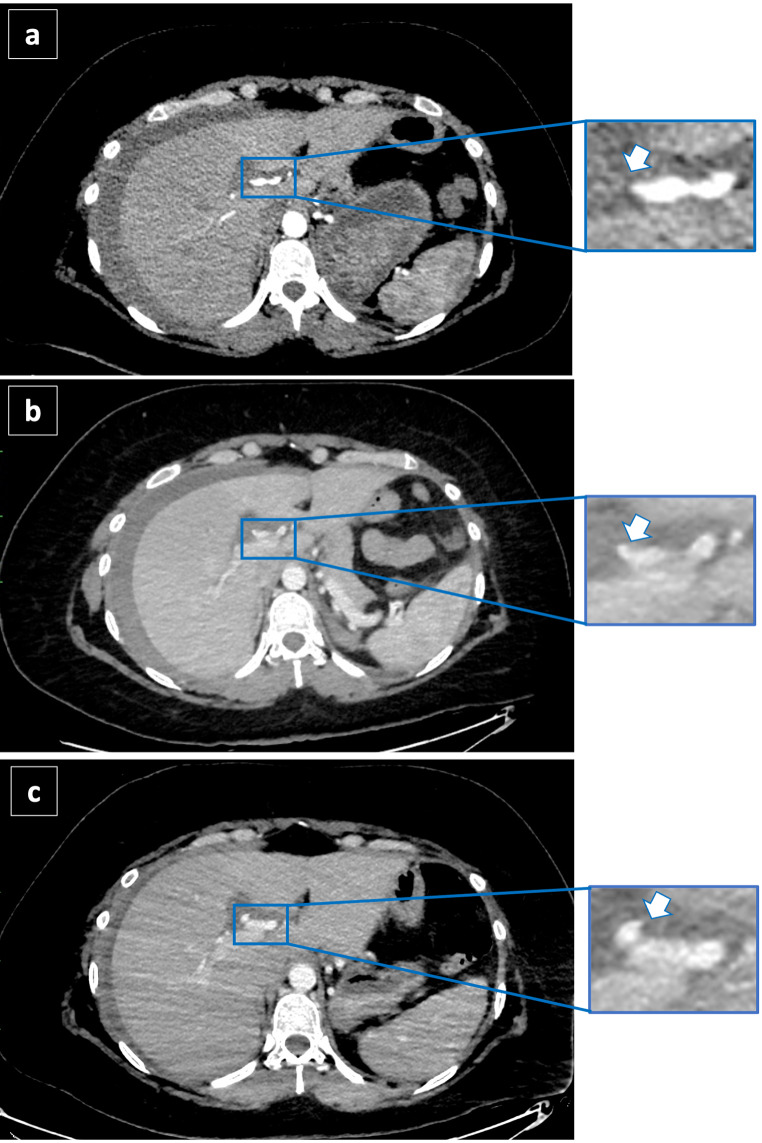
Contrast - enhanced CT after iodinated contrast agent injection during arterial phase on arrival at the Emergency Department (**a**) 20-hours after admission (**b**) and 5 days after admission at the Emergency Department (**c**). The contrast leakage (arrow) at the level of hepatic artery becomes progressively larger from 2 mm (**a**), up to 4 mm (**b**) and 7 mm (**c**) and becomes progressively more evident

A 36-year-old woman with a history of obesity and cholecystolithiasis complicated with acute pancreatitis was admitted to the hospital for an elective laparoscopic cholecystectomy (LC). The surgeons reported that during the dissection of the Calot’s Triangle (bounded by the gallbladder wall, cystic duct, and common hepatic duct), a massive bleeding occurred, forcing the surgeon to convert to open cholecystectomy. The source of bleeding was identified, and the surgeon reported that he sutured the origin of the cystic artery with surgical thread and applied 2 clips to ensure optimal hemostasis. Cholecystectomy was then completed. The patient spent other two days at the hospital in good clinical conditions except for a decrease of hemoglobin (Hb) level, from 11.4 g/L immediately after the surgery to 8.7 g/dL. Six days after the discharge she suffered a transient loss of consciousness followed by weakness, nausea and chest pain and she was taken to the hospital in emergency and admitted to the surgery department. The Hb level at admission was 9.4 g/l. An abdominal CT scan was performed immediately and then repeated after 20 h, revealing signs of hemoperitoneum, without identifying any source of bleeding. After 6 days the patient was discharged in good clinical conditions although laboratory tests showed decreasing hemoglobin levels, requiring blood transfusions (6.8 g/L the second day of hospitalization, before blood transfusion, and 8.2 g/L after blood transfusion). Before the discharge, another CT scan was performed and, again, no sign suggestive for active bleeding was identified. Twenty-four hours after discharge she experienced a fatal cardiocirculatory arrest at home. An autopsy was requested by the Public Prosecutor to determine the cause of death. Extensive hemorrhagic infiltration of the peritoneum and 5 surgical clips at the hepatic hilum were observed: 2 clips adjacent to the suture used for ligating the cystic artery, and 3 clips on the right hepatic artery, that should not be present in a routine cholecystectomy (Fig. 1). A post-mortem review of the CT scan images was then performed. The first abdominal CT scan images, taken 10 days after the cholecystectomy, revealed a subtle extra-vasal leakage of contrast medium of 2 mm from the right hepatic artery (Fig. 2a). The second CT scan, taken 20 h later, showed a larger leakage of 4 mm (Fig. 2b). The leakage was increased in size (7 mm) at the CT scan performed after 15 days from surgery (Fig. 2c). These findings allowed a post-mortem diagnosis of ruptured PA of right hepatic artery, developed in the site of application of surgical clips. The cause of death was determined to be hemorrhagic shock resulting from massive hemoperitoneum.

## Discussion

In this case, the medico-legal interest is to evaluate the causal relationship between the cholecystectomy and the death, given that no diagnosis was made during hospitalization despite the progressive anemia of the patient after surgery and the signs of hemoperitoneum at CT images. Besides the 2 clips used for ligating the cystic artery, macroscopic analysis of hepatic hilum revealed the presence of 3 surgical metal clips on the right hepatic artery, that should not be present in a routine cholecystectomy. Review of abdominal CT scan images allowed visualization and localization of the source of the fatal intra-peritoneal bleeding, that was an iatrogenic right hepatic artery pseudoaneurysm, close to the 3 clips. CT images also showed a rapid increasing in size of the PA: at 10 days from LC, it doubled its size in 20 h (from 2 to 4 mm), and then it was again almost doubled (7 mm) 15 days after surgery, the day before the death.

LC is the gold standard treatment for symptomatic cholelithiasis and one of the most performed abdominal operations worldwide. Nevertheless, it is commonly complicated with biliary and vascular injuries because of the proximity of these structures to the gallbladder and the complexity of Calot’s Triangle dissection. Pseudoaneurysms (PA), also known as “false aneurysm”, are rare but life-threatening events following LC, with an incidence between 0.06% and 0.6% [1, 2]. PA occur due to leakage from an injured artery into the surrounding tissues forming a cavity outside the artery. They can be distinguished from hematoma as they continue to communicate with the arterial lumen resulting in a high-pressure cavity with the risk of rupture [3, 4], that occurs in 80% of cases [5]. Lampropoulos et al. [6] in their 26-year period review of literature (1994–2000) found that the hepatic artery is the most common injured vessel during cholecystectomy (70%), followed by the cystic artery (19%). In most cases injuries to these vessels are due to dissection procedure or trocar insertion (66%) and the most common symptoms were gastrointestinal bleeding (74%) and abdominal pain (61%) few weeks after the procedure. Diagnosis of PA may be difficult, and 80% of them present with life-threating rupture as the initial clinical event [7]. Diagnosis of right hepatic artery PA is normally based on in vivo “dynamic” imaging (CT angiography), which provides accurate information regarding the anatomy of visceral aneurysms and delineates the extent of extravasation and hemoperitoneum as well as demonstrates any associated intraperitoneal or retroperitoneal pathology. Treatment options depend on the type of vessel injured and include trans-arterial embolization, surgical repair for hepatic artery and clipping for cystic artery. These repairing techniques have high success rate and low mortality [6].

In forensic literature, cases of death following PA rupture regard almost exclusively injuries of femoral artery in injected drug abusers [8, 9], where autoptic diagnosis is oriented by superficial skin ulcerations in continuity with the artery wall. The post-mortem diagnosis of visceral PA is challenging and rarely reported as post-mortem finding, since it requires a correspondence between in vivo imaging, that represents the diagnostic gold standard, and the post-mortem localization of the vascular injury [10]. In fact, as the PA forms in a virtual cavity, the autopsy alone cannot identify the progression of the PA.

The innovation of this report is to present an iconographic correlation between in vivo imaging and autopsy data, allowing for the tracing of the PA’s origin to the wall weakening caused by the placement of 3 clips on the right hepatic artery. The slow bleeding that originated from the surgical site led to death 16 days after the procedure, confirming the causal relationship between the procedure and the death.

It is not uncommon for intra-operative vascular injuries to be identified for the first time by the forensic pathologist [11], who must correctly identify the injured vessel and the timing of the injury, which is not always possible through a post-mortem evaluation. As previously reported in other cases of suspected medical liability [12], the present case underscores the importance of carefully reviewing antemortem imaging to guide autopsy and integrate radiological and autoptic information for reconstructing the events.

## Data Availability

N/A.
